# Risk Factors for *Clonorchis sinensis* Infection in Residents of Binyang, Guangxi: A Cross-Sectional and Logistic Analysis Study

**DOI:** 10.3389/fpubh.2021.588325

**Published:** 2021-05-05

**Authors:** Meng Xu, Yanyan Jiang, Jianhai Yin, Shengkui Cao, Yujuan Shen, Jianping Cao

**Affiliations:** ^1^National Institute of Parasitic Diseases, Chinese Center for Disease Control and Prevention, Chinese Center for Tropical Diseases Research, Shanghai, China; ^2^NHC Key Laboratory of Parasite and Vector Biology, Shanghai, China; ^3^World Health Organization Collaborating Centre for Tropical Diseases, Shanghai, China; ^4^National Center for International Research on Tropical Diseases, Shanghai, China; ^5^School of Global Health, Chinese Center for Tropical Diseases Research, Shanghai Jiao Tong University School of Medicine, Shanghai, China

**Keywords:** *Clonorchis sinensis*, risk factor, prevalence, logistic analysis, Guangxi

## Abstract

**Background:** Clonorchiasis is a serious food-borne parasitic disease caused by *Clonorchis sinensis* infection. *C. sinensis*, a major fish-borne trematode, is a known causative agent of cholangiocarcinoma. The risk factors for *C. sinensis* infection include individual eating behaviors and environmental factors. In this study, we evaluated the *C. sinensis* infection rate and the associated risk factors among residents in Binyang County, Guangxi, China.

**Methods:** In 2016 and 2017, five villages from Binyang, Guangxi were selected by multistage cluster random sampling for a cross-sectional study. A modified Kato-Katz thick smear method was used to examine *C. sinensis* eggs in fecal samples in triplicate (three smears for each sample). Both uni-variate and multi-variate logistic regression analyses were carried out to identify the risk factors for *C. sinensis* infection.

**Results:** A total of 1,977 fecal samples were collected from villagers in the investigated areas. The overall infection rates of *C. sinensis* in Binyang County was 20.49% (405/1,977). The mean age of participants was 39.42 ± 23.48 (range: 3–89 years old), and the highest infection rate (33.72%) was seen in the age group of 40-49 years old, followed by those aged 50–59 (31.83%). Multi-variate logistic regression analysis showed that higher infection rates were significantly associated with males (*aOR* = 6.51, *95% CI* = 4.67–9.08), Zhuang (*aOR* = 2.41, *95% CI* = 1.62–3.59), ages (*aOR* = 33.51, *95% CI* = 10.13–110.86), frequency of raw fresh fish consumption (*aOR* = 14.56, *95% CI* = 9.80–21.63), and close contact with cats and dogs (*aOR* = 1.53, *95% CI* = 1.02–2.30). Occupations and education levels showed no significant association with *C. sinensis* infection (*P* > 0.05).

**Conclusions:** High levels of *C. sinensis* infection were observed among residents in Binyang County, Guangxi. Intervention strategies should be strengthened among the investigated population at high risk, such as males, Zhuang and older individuals, especially those who frequently eat raw fresh fish. In addition, the individuals contacting with cats and/or dogs were observed to have significantly higher infection rate of *C. sinensis* than those having no contact with cats and dogs. The association between contacting with cats and/or dogs and *C. sinensis* infection needs to be explored and confirmed in the future study by more epidemiological investigations of human *C. sinensis* infection from different areas.

## Background

Clonorchiasis is a serious food-borne trematodiasis. Adult flukes inhabit the bile ducts of the definitive host (1) and cause complications such as biliary fibrosis, cholangitis, cholecystitis, liver fibrosis, cholelithiasis, and liver cirrhosis (2–5). In 2009, *C. sinensis* was classified as a group I biological carcinogen for cholangiocarcinoma by the International Agency for Research on Cancer (IARC) (6, 7).

Clonorchiasis is predominantly endemic in China, Korea, northern Vietnam and eastern Russia. It poses a considerable public health problem and disease burden, and could cause a loss of 275,370 DALYs (3, 8, 9). It was estimated that 15 million people were infected with *C. sinensis* globally in 2004, of which about 85% were in China (9, 10). Clonorchiasis cases were concentrated in two regions of China: the southeast (i.e., Guangdong and Guangxi) and the northeast (i.e., Heilongjiang and Jilin) (9, 11). In 1985, clonorchiasis was prevalent in 23 counties (12), and increased to 60 counties in 2014 (13). Three national parasite surveys showed the prevalence to be increasing in Guangxi: 1.39% in 1988–1992, 4.01% in 2002–2004, and 9.62% in 2014–2015 (14, 15). Shu et al. (16) found that the positive rates of *C. sinensis* IgG antibody among humans in Guangxi were 21.83% (2016), 29.88% (2017), and 36.48% (2018). At present, clonorchiasis remains a significant public health problem in Guangxi, in which there is a high risk of *C. sinensis* infection and a large number of infected people (11).

Eating raw fresh fish contaminated with *C. sinensis* metacercariae is believed to be the major route for *C. sinensis* infection (10, 17, 18). Understanding of the prevalence and risk factors of clonorchiasis is an important step to improve or set up effective control programs to enhance the health status of residents. In this study, we examined the prevalence of *C. sinensis* infection by the Kato-Katz method and collected socio-demographic information and possible risk factors via questionnaire for multi-variate logistic regression analysis.

## Methods

### Area Surveyed

**Figure 1:**
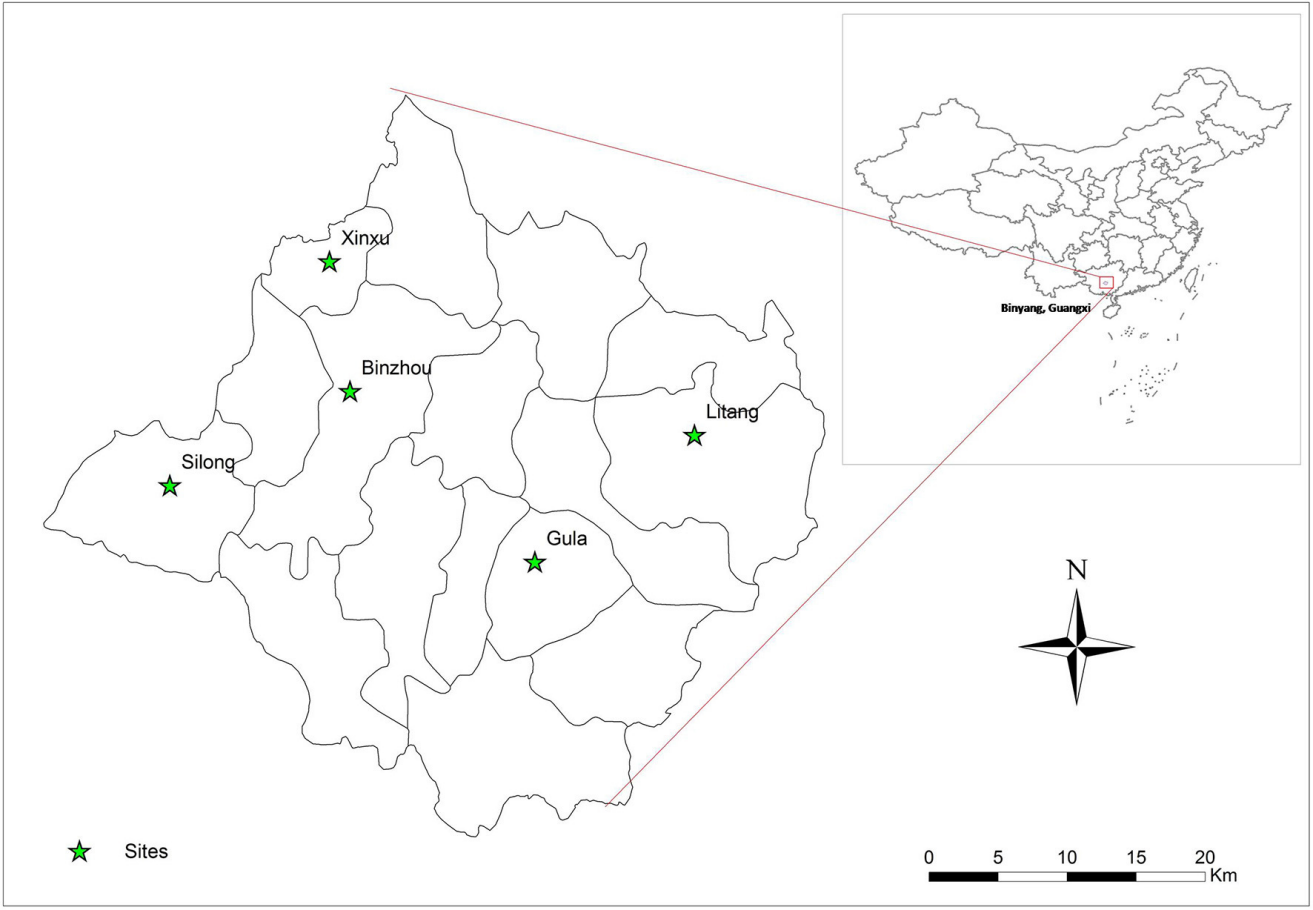
Surveyed sites: five villages in Binyang, Guangxi.

A cross-sectional study of *C. sinensis* infection was undertaken from 2016 to 2017 in Binyang County, Guangxi Zhuang Autonomous region, China. This county is located in the Pearl River Basin in Southern China, and has 16 towns with a population of ~1.1 million people. Five villages were randomly selected from five towns (one each) by multistage cluster. In each survey village, about 400 households were selected and one member was randomly enrolled from each household for this survey. The five surveyed towns are shown in Figure 1 by ArcGis 10.1 (Figure 1).

### Sample Size

The primary objective of this study was to estimate the prevalence of clonorchiasis and generate hypotheses about possible risk factors of *C. sinensis* infection among the residents. To address this, the required sample size was calculated using the formula: $n=\frac{z_{\alpha }^{2}\times pq}{d^{2}}$ (19) with a 95% confidence interval, an estimated infection prevalence of 24.00% (20), and tolerance error *d* = 0.1 ^*^ *p* = 0.024. The resulting sample size was 1,217 participants. Taking into account the failure to submit a stool sample for an estimated 10% of participants, the sample size increased to 1,339 individuals after adjustment.

### Participant Recruitment

Residents from the five sampling sites formed the target population in this study. The inclusion criteria were: age over 3 years old; agreed to take part in the study; and a resident for at least 6 months. The medical staff at the local Center for Disease Control and Prevention took part in the participant recruitment.

### Questionnaire, Fecal Sample Collection, and Laboratory Examination

A questionnaire was used to interview all participants to collect socio-demographic information and possible *C. sinensis*-related behaviors. The information included name, gender, ethnicity, birth date, name of school or workplace, phone number, address, occupation, and education level. The *C. sinensis* infection-related behaviors included the habit and the frequency of raw fresh fish consumption as well as contact with a cats and/or dogs. Each investigator of this study received uniform training on the importance of the study and on how to correctly collect the data from participants before beginning the investigation. During questionnaire collection, investigators were responsible for checking and revising missing items and logical errors by asking the participants. Quality control was performed throughout the process by investigators of the study.

Each participant recevied a labeled sample plastic container for stool collection (≥5 g) for this investigation. The collected fecal samples were transferred to a local laboratory within 4 h and stored at 4°C before modified Kato-Katz thick smear method. The modified Kato-Katz method using ~41.7 mg templates was employed to examine for the presence of *C. sinensis* eggs in the fecal sample in triplicate (three smears for each sample) (21).

### Statistical Analyses

All of the information, including stool examination and questionnaire data were entered in an Excel file by one investigator and checked by another. Analysis was conducted using SPSS (version 20.0). Mean and standard deviation were used to describe the data. Uni-variate logistic regression analysis was carried out to evaluate the association between *C. sinensis* infection and each of questionnaire items, considering *P* < 0.05 and a 95% confidence interval (95% *CI*). The variables, which were significantly associated with *C. sinensis* infection (*P* < 0.05), were all entered into multi-variate logistic regression for final analysis.

## Results

### Characteristic of the Study Population

A total of 2,056 individuals participated in this study. Of these, 79 individuals, including 76 children below 3 years old and three paiticipants (their was 9, 29, 56 years, respectively), were excluded due to none of stool samples. Out of 1977 (95.91%) participants, 993 (50.23%) were male and 984 (49.77%) were female. The age of the 1977 participants was 39.42 ± 23.48 years (range: 3–89 years old) (Table 1).

**Table 1:** Socio-demographic characteristics of participants (*n* = 1,977).

**Variables**	**Number**	**Rate (%)**
**Age**		
3–9	380	19.22
10–19	174	8.80
20–29	114	5.77
30–39	225	11.38
40–49	261	13.20
50–59	355	17.96
60–69	284	14.37
70–89	184	9.31
**Gender**		
Female	984	49.77
Male	993	50.23
**Ethnic group**		
Han	1,763	89.18
Zhuang	214	10.82
**Education**		
Primary school and below	965	48.81
Junior high school	858	43.40
Senior high school	86	4.35
College degree or above	68	3.44
**Occupation**		
Non–farmer	697	35.26
Farmer	1,280	64.74
**Raw fresh fish consumption**		
0 time/year	1,487	75.21
1 time/year	288	14.57
1–4 times/month	202	10.22
**Contacting with cats or dogs**		
No	1,774	89.73
Yes	203	10.27

### Prevalence of *C. sinensis*

In this study 405 were egg-positive for *C. sinensis*, making the overall prevalence of *C. sinensis* infection 20.49% (405/1,977). The positivity rate in males (33.43%, 332/993) was 6.27 times higher than in females (7.42%, 73/984) (*cOR* = 6.27, *95% CI* = 4.77–8.23). The highest positivity rate in the age group of 40–49 years old (33.72%, 88/261), which was 47.82 times higher than in the age group of 3–9 years old(*cOR* = 47.82, *95% CI* = 17.28–132.34). The positivity rate was 2.84 times higher in Zhuang people (38.79%, 83/214) than in Han people (18.26%, 322/1,763) (*cOR* = 2.84, *95% CI* = 2.10–3.83). The highest positivity rate occurred in those with senior high school degree (30.23%, 26/86), which was 3.37 times higher than those with primary school and below (*cOR* = 3.37, *95% CI* = 2.04–5.56). The participants eating raw fresh fish 1–4 times one month had the highest positivity rate of 64.36% (130/202), which was 19.00 times higher than participants who never ate raw fresh fish (*cOR* = 19.00, *95% CI* = 13.53–26.70) (Table 2).

**Table 2:** Uni-variate and multi-variate analysis of risk factors associated with *C. sinensis* infection in Binyang, Guangxi (*n* = 1,977).

**Characteristic**	**Positive no. (%)**	**Uni-variate analysis**	**Multi-variate analysis**
		***cOR (95% CI)***	***aOR (95% CI)***
**Age**			
3–9	4/380 (1.05)	1	1
10–19	11/174 (6.32)	6.34 (1.99–20.22)^*^	6.55 (1.97–21.78)^*^
20–29	20/114 (17.54)	20.00 (6.68–59.91)^*^	10.68 (3.00–38.03)^*^
30–39	62/225 (27.56)	35.76 (12.79–99.92)^*^	20.79 (6.32–68.39)^*^
40–49	88/261 (33.72)	47.82 (17.28–132.34)^*^	33.51 (10.13–110.86)^*^
50–59	113/355 (31.83)	43.89 (15.98–120.53)^*^	24.66 (7.50–81.07)^*^
60–69	65/284 (22.89)	27.90 (10.03–77.63)^*^	16.54 (5.05–54.14)^*^
70–89	42/184 (22.83)	27.80 (9.79–78.94)^*^	19.07 (5.74–63.33)^*^
**Gender**			
Female	73/984 (7.42)	1	1
Male	332/993 (33.43)	6.27 (4.77–8.23)^*^	6.51 (4.67–9.08)^*^
**Ethnic group**			
Han	322/1,763 (18.26)	1	1
Zhuang	83/214 (38.79)	2.84 (2.10–3.83)^*^	2.41 (1.62–3.59)^*^
**Education**			
Primary school and below	110/965 (11.40)	1	1
Junior high school	249/858 (29.02)	3.18 (2.48–4.07)^*^	1.07 (0.75–1.54)
Senior high school	26/86 (30.23)	3.37 (2.04–5.56)^*^	1.13 (0.59–2.17)
College degree or above	20/68(29.41)	3.24 (1.85–5.66)^*^	1.02 (0.44–2.37)
**Occupation**			
Non–farmer	56/697 (8.03)	1	1
Farmer	349/1,280 (27.27)	4.29 (3.18–5.79)^*^	1.18 (0.68–2.02)
**Raw fresh fish consumption**			
0 time/year	129/1,487 (8.68)	1	1
1 time/year	146/288 (50.69)	10.82 (8.07–14.51)^*^	4.22 (3.02–5.90)^*^
1–4 times/month	130/202(64.36)	19.00 (13.53–26.70)^*^	14.56 (9.80–21.63)^*^
**Contacting with cats or dogs**			
No	334/1,774 (18.83)	1	1
Yes	71/203 (34.98)	2.32 (1.70–3.17)^*^	1.53 (1.02–2.30)^*^

*CI, confidence interval; cOR, crude odds ratio; aOR, adjusted odds ratio*.

* *P < 0.05*.

### Correlation Between *C. sinensis* Infection and Possible Risk Factors

Based on the uni-variate analysis, age, gender, ethnic group, education level, occupation, raw fresh fish consumption, and contact with cats or dogs were all significantly associated with *C. sinensis* infection (*P* < 0.05). The following variables were risk factors for infection with *C. sinensis:* age ≥20 years old, Zhuang ethnicity, high level of education, farmers, raw fresh fish consumption (including eating raw fresh fish once a year and 1–4 times a month) and contacting with cats or dogs. The *cOR* value and 95% *CI* are shown in Table 2.

In the multi-variate model, age, gender, ethnic group, raw fresh fish consumption, and contacting with cats or dogs were retained as predictors, and they were all significantly associated with *C. sinensis* infection. Being male increased the risk of *C. sinensis* infection 6.51-fold compared to being female (*aOR* = 6.51, 95% *CI* = 4.67–9.08). Participants who were Zhuang were 2.41 times more likely to be infected than those who were Han (*aOR* = 2.41, 95% *CI* = 1.62–3.59). Those who contacted with cats and/or dogs were 1.53 times more likely to be infected with *C. sinensis* than those who did not (*aOR* = 1.53, 95% *CI* = 1.02–2.30). Participants who ate raw fresh fish once a year were 4.22 times more likely to be infected than those who did not eat raw fresh fish (*aOR* = 4.22, 95% *CI* = 3.02-5.90). Similarly, participants who ate raw fresh fish 1–4 times a month were 14.56 times more likely to be infected than those who did not eat raw fresh fish (*aOR* = 14.56, 95% *CI* = 9.80–21.63). In the different age groups, the highest prevalence of *C. sinensis* was in the 40–49 age group, where the rate was 33.51 times that of the 3–9 age group (*aOR* = 33.51, 95% *CI* = 10.13–110.86). The variables of occupation and education level showed no significant association with *C. sinensis* infection (*P* > 0.05).

## Discussion

The results of this study show that the overall prevalence of *C. sinensis* in Binyang was 20.49%, lower than a previous investigation (24.73%) conducted in 2001–2006 (20). In spite of this, Binyang is still a heavy clonorchiasis endemic area according to modern parasitology infection grades (22).

The aforementioned studies revealed that the higher prevalence of *C. sinensis* infection in fish [8.46–43.88% (20, 23)], cats (over 50%) and dogs (30%) in the endemic areas of higher clonorchiasis prevalence (20, 24). Previous studies found that eating raw fresh fish was a major risk factor for *C. sinensis* infection in humans (10, 17, 18). A similar result was observed in our study, and infection risk increased with frequency of raw fresh fish consumption. This research also found that participants who contacted with cats or dogs were 1.53 times more likely to be infected than their counterparts. Locally, the main food source for cats is raw fresh fish. The cats' tongue may transfer *C. sinensis* metacercariae to its fur, for it prefers to lick their fur to remove odors and dirt. What's more, the fur of cats and/or dogs may get metacercariae for exposuring to enviroments contaminated by metacercariae from the slaughter of fishes. The one who eat without washing their hands after play with cats/dogs may be infected. The association between contacting with cats or dogs and *C. sinensis* infection needs to be explored and confirmed in the future study by more epidemiological investigations of human *C. sinensis* infection from different areas. What's more, the future study focus on the presence and the survival time of *C. sinensis* metacercaria in the fur of cats or dogs.

The present study showed that the likelihood of *C. sinensis* infection was 6.51 times higher in males than in females. Similar results were also observed in some previous studies conducted in Guangxi (25–27) and other countries (10, 17, 28). The probable reason for this might be the greater involvement of males in outdoor activities such as dining together. Meanwhile, the higher prevalence in Zhuang people was in agreement with another report in Guangxi (29). Usually, the Zhuang people had significantly higher alcohol consumption than Han people (29). Drinking alcohol is always accompanied with eating raw fresh fish (9, 17), leading to an important risk factor for *C. sinensis* infection in this population.

In this survey, *C. sinensis* infection was seen in all age groups. The prevalence reached a maximum in the 40–49 age group, followed by the 50–59 age group. The infection peak occurred in the 40–49 age group, which was consistent with the result in an epidemiological study in Nanning City, Guangxi in 2010 (27). Similar findings in other areas were also reported and showed with a peak in the age groups of 40–49 or 50–59 years old, then decreasing afterwards (9, 30–32). In the endemic area, it was observed that there was a positive relationship beween infection rates and ages less than about 50 years old, which may be an accumulation effect of reinfection or superinfection with age, because previous infection has little protective impact on reinfection or superinfection in humans (33). The lower infection in the older groups was also observed in previous investigations (27, 30–32), suggesting a shorter lifespan of residents with clonorchiasis compared with those who are uninfected (34). This result indicated that education is necessary for all age groups to change local residents' habit of eating raw fresh fish to decrease *C. sinensis* infection. Intervention strategies, supervision and control of the infection status of *C. sinensis* metacercariae among fish markets and restaurants should be strengthened in the investigated areas.

In uni-variate logistic regression analyses, the prevalence of *C. sinensis* infection among farmers was significant higher than in non-farmers. Similar results were found in a study in Korea (35). Across the different education levels, those with primary school education had significantly lower prevalence compared to those with junior high school, senior high school and a college degree or above in uni-variate logistic regression analyses. The result was in contrast to studies in North Vietnam and Korea, which found higher prevalence in lower educational levels, mainly at elementary school level (17, 36). The probable reason for this difference might be the variation in environment and living habits of the study participants and the different factors of *C. sinensis* infection studied by different researchers.

In this study, there were several limitations which should be considered. Since infection-related behaviors were self-reported, recall bias may exist. Some efforts were made to minimize this during the questionnaires; for example, investigators used a local recipe with raw fresh fish to ascertain whether they ate raw fresh fish and the frequency of doing so. To avoid bias from investigators, each investigator of this study received uniform training before the study began, which ensured that the same inquiry method was used to obtain accurate and reliable information. Furthermore, this study used a modified Kato-Katz thick smear method, the gold standard for diagnosis of *C. sinensis* infection and widely used because of its simplicity, low cost, and the ability to quantify infection intensity (37). However, the prevalence of *C. sinensis* among residents may have been underestimated, since this method has a low sensitivity and a high missing diagnosis rate (21). To correctly investigate the prevalence and assess the risk factors for clonorchiasis, an auxiliary diagnostic method such as PCR or ELISA could be used (38, 39). Repeated Kato-Katz smears from multiple stool samples could also be used to improve diagnostic accuracy (38).

## Conclusions

High levels of *C. sinensis* were recorded among residents in Binyang, Guangxi. In this study, we found that the risk factors for *C. sinensis* infection were being males, Zhuang, the older, and frequency of raw fresh fish consumption. In addition, we found that the prevalence of *C. sinensis* infection among individuals who contacted with cats and/or dogs was significantly higher than in those who did not. The association between contacting with cats and/or dogs and *C. sinensis* infection needs to be explored and confirmed in the future study by more epidemiological investigations of human *C. sinensis* infection from different areas. These data make useful contributions to infection risk prevention interventions carried out in Binyang and help to develop culturally suitable and effective clonorchiasis prevention and control.

## Data Availability Statement

The datasets generated and/or analyzed during the current study are not publicly available to protect participant confidentiality.

## Ethics Statement

Ethical approval for the questionnaires and study procedures involving human stool sample collection and examination was obtained from the Ethics Committee of the National Institute of Parasitic Diseases, Chinese Center for Disease Control and Prevention, China (No. 201401). All participants and the guardians of child participants were informed of the study objectives, procedures, and potential risks. Meanwhile, written informed consent was personally signed by each adult participants, and parents or guardians were asked to provide written consent on behalf of child participants at sampling sites before collection of fecal samples involved in our study. The personal information of all participants has remained confidential.

## Author Contributions

YS and JC designed this study. MX, SC, and YJ performed the experiments. MX and JY analyzed the data. MX wrote the manuscript and prepared the tables and figures. YS and JC revised the manuscript. All authors read and approved the manuscript.

## Conflict of Interest

The authors declare that the research was conducted in the absence of any commercial or financial relationships that could be construed as a potential conflict of interest.
